# Biological Activity Profiles of Multitarget Ligands from X-ray Structures

**DOI:** 10.3390/molecules25040794

**Published:** 2020-02-12

**Authors:** Christian Feldmann, Jürgen Bajorath

**Affiliations:** Department of Life Science Informatics, B-IT, LIMES Program Unit Chemical Biology and Medicinal Chemistry, Rheinische Friedrich-Wilhelms-Universität, Endenicher Allee 19c, D-53115 Bonn, Germany; cfeldmann@bit.uni-bonn.de

**Keywords:** complex X-ray structures, multitarget ligands, promiscuity, compound databases, biological screening, medicinal chemistry, activity data, ligand activity profiles, data consistency

## Abstract

In pharmaceutical research, compounds with multitarget activity receive increasing attention. Such promiscuous chemical entities are prime candidates for polypharmacology, but also prone to causing undesired side effects. In addition, understanding the molecular basis and magnitude of multitarget activity is a stimulating topic for exploratory research. Computationally, compound promiscuity can be estimated through large-scale analysis of activity data. To these ends, it is critically important to take data confidence criteria and data consistency across different sources into consideration. Especially the consistency aspect has thus far only been little investigated. Therefore, we have systematically determined activity annotations and profiles of known multitarget ligands (MTLs) on the basis of activity data from different sources. All MTLs used were confirmed by X-ray crystallography of complexes with multiple targets. One of the key questions underlying our analysis has been how MTLs act in biological screens. The results of our analysis revealed significant variations of MTL activity profiles originating from different data sources. Such variations must be carefully considered in promiscuity analysis. Our study raises awareness of these issues and provides guidance for large-scale activity data analysis.

## 1. Introduction

Multitarget activity of small molecules continues to be a much debated topic in drug discovery [1,2]. Current views are that many pharmaceutically relevant compounds elicit therapeutic effects in vivo through interactions with multiple targets, a phenomenon referred to as polypharmacology [1,2,3]. On the other hand, multitarget activity, also termed promiscuity [4], is responsible for undesired side effects. Importantly, a general promiscuity assumption is difficult to prove and generalise beyond individual case studies. However, systematic analysis of in vitro compound activity data can be carried out to estimate promiscuity on a large scale [3,4,5]. Activity data analysis is inevitably affected by data incompleteness, since available small molecules will hardly ever be tested against all potential pharmaceutical targets [5]. Furthermore, activity data analysis is influenced by assay and data confidence criteria [6] as well as potential experimental artifacts [7]. However, given the very large volumes of compound activity data that have accumulated in the public domain [8,9], such analyses yield data-driven assessments of molecular promiscuity that go far beyond subjective views and intuitive expectation values. For example, on the basis of currently available activity data, bioactive compounds are generally less promiscuous than often assumed [5,10]. On the other hand, large numbers of compounds with confirmed activity against proteins from different families or classes have also been identified on the basis of assay data [11] and publicly available X-ray structures of ligand-target complexes [12,13]. Hence, small molecules are often capable of interacting with distantly or unrelated proteins; a facet of promiscuity that is of particular interest for further investigation, both from a practical and basic research perspective.

Another important factor affecting compound promiscuity analysis is activity data consistency across different sources. For example, varying experimental settings are expected to modulate activity readouts. In addition, alternative data curation and confidence criteria are likely to yield different compound activity annotations, depending on the source from which they are retrieved. So far, however, such issues have only been little investigated in the context of promiscuity analysis. Currently, there is no comprehensive assessment of data consistency in promiscuity exploration available. Therefore, we have carried out a systematic analysis to determine and compare activity profiles of known promiscuous compounds on the basis of high-throughput screening data and activity annotations from medicinal chemistry sources. Since our analysis critically depended on small molecules with confirmed multitarget activity, we initially identified compounds that were available in X-ray structures of complexes with different targets. For these structurally confirmed multitarget ligands (MTLs), assay data and activity annotations were collected, and their current activity profiles determined. Our study and the results are presented in the following. Taken together, the findings revealed that MTLs have in part strongly varying profiles and that activity data from different sources often have limited overlap and consistency. The latter observation is not necessarily a consequence of experimental variance but often due to different data curation schemes. Hence, from several points of view, care must be taken to consider activity data consistency in estimating compound promiscuity. 

## 2. Results and Discussion

### 2.1. Analysis Concept 

The analysis aimed to determine activity profiles of MTLs and investigate their consistency on the basis of different activity data sources. Activity profiles were defined as the union of all available positive and negative target annotations from a given data source. For the analysis, the availability of confirmed MTLs was essential as a starting point. We reasoned that promiscuous compounds can be most confidently identified on the basis of X-ray data that confirm the presence of different ligand-target interactions directly. Therefore, we initially searched X-ray structures of complexes for ligands with defined chemical characteristics bound to different targets. Although the identification was laborious, it provided MTLs at a high level of confidence. Subsequent activity data analysis was centered on these compounds and emphasis was put on the question of how MTLs from X-ray structures might act across biological screens. Focusing on screening data initially made it also possible to take target test frequencies into account, which provide important information for activity assessment. For comparison, activity data from medicinal chemistry sources were used to evaluate activity profiles. Hence, the analysis scheme was focused on the evaluation of activity data consistency from different viewpoints. We note that specific structural features that may be generally responsible for ligand promiscuity are currently unknown (if they exist). 

### 2.2. Confirmed Multitarget Ligands 

**Figure 1:**
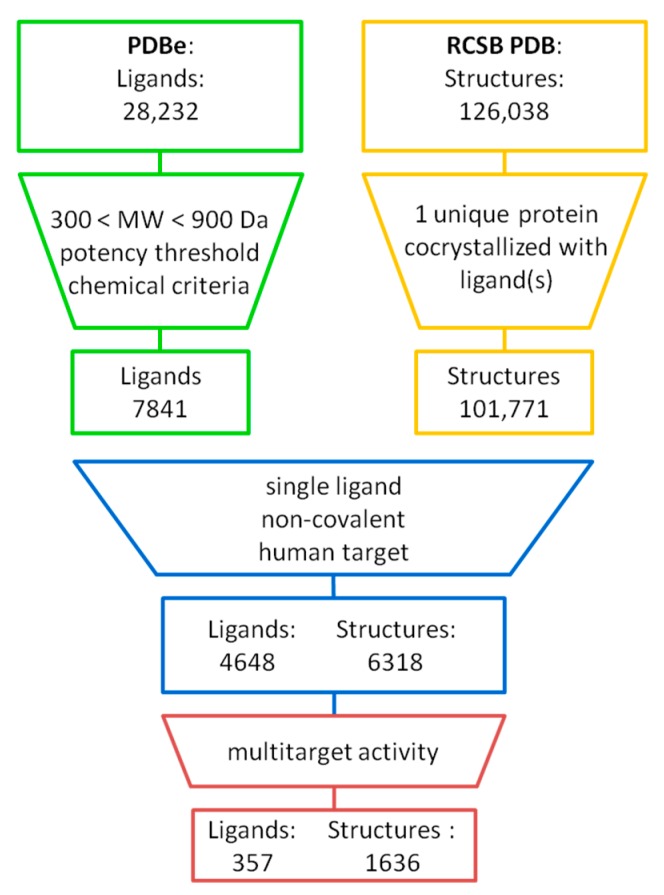
Ligands from X-ray structures. From Protein Data Bank (PDB) entries, unique ligands were extracted and filtered for molecular weight, potency annotations, and (bio-)chemical characteristics (see Materials and Methods for details). Only human proteins complexed with single non-covalent ligands were further considered. Qualifying X-ray ligands in complexes with different targets were identified, yielding 357 MTLs.

Figure 1 summarises the identification of MTLs from X-ray structures (further details are provided in Materials and Methods). X-ray ligands representing pharmaceutically relevant synthetic compounds were selected and structures with human proteins containing them. The pre-selected 4648 ligands found in 6318 complex structures were then searched for compounds in complexes with different targets. The search identified 357 MTLs occurring in 1636 X-ray structures. These MTLs included 176 compounds found in complexes with proteins from different families. MTLs interacted with 2–19 targets, with a median of 2 targets per compound.

### 2.3. Multitarget Ligands in Biological Screens 

The 357 MTLs were mapped to 2686 qualifying high-throughput screens with human targets available in PubChem (see Materials and Methods), the major public repository of biological screening data. These screens included 2576 single-target and 110 target panel assays. All targets were clearly defined. In these PubChem assays, 126 of our MTLs from X-ray structures were detected, which provided the basis for our subsequent analysis. 

**Figure 2:**
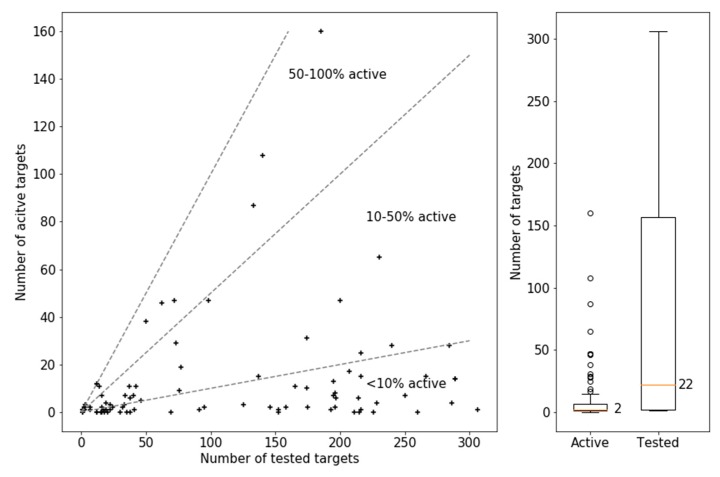
Activity annotations of MTLs in PubChem. The scatter-plot on the left compares the number of targets against which MTLs were tested (tested targets) and active (active targets). Each data point represents an MTL. Dashed lines mark similar ratios of active over tested targets. The boxplots on the right report the distributions of the number of tested targets (corresponding to test frequency) and active targets per MTL. The reported median is represented by an orange vertical line. The lower and upper boundaries of the boxes indicate the lower and upper quartile, respectively. The length of the whiskers corresponds to 1.5-fold of the interquartile range. Circles above or below the whiskers represent statistical outliers.

Figure 2 reports the test frequency of these MTLs in PubChem assays and their activity. The boxplot on the very right shows that MTLs were in part extensively assayed including 39 MTLs tested in 100 to more than 300 assays, with a median test frequency of 22 assays per compound. However, the other boxplot reveals that their activity was generally limited, with a median of 2 active targets per MTL. There were only 3 compounds with excessive activity against nearly 100, more than 100, and more than 150 targets, respectively. Although these compounds formed well-defined crystallographic interaction with different targets, their excessive activity might at least partly be attributed to undesired reactivities under assay conditions and ensuing artificial activity readouts. However, the scatter plot in Figure 2 shows that MTLs with excessive activity in screening assays were notable exceptions. In fact, the majority of confirmed MTLs were only active against fewer than 10% of the targets they were tested against. These also included 5 MTLs that were evaluated in 100 or more assays and were consistently inactive. By contrast, only a few MTLs were active against more than half of the targets they were tested against.

Taken together, these observations revealed an important point. Confirmed MTLs from X-ray structures were generally far from being highly promiscuous, although they were often tested against many targets. These findings were consistent with the ability of most MTLs to engage in well-defined -rather than ‘unspecific’- interactions with a confined number of targets. For such compounds, meaningful activity analysis can be carried out across different data sources. 

### 2.4. Target Annotations from Medicinal Chemistry

The 126 MTLs detected in PubChem assays were also searched in ChEMBL, the major source of compounds and activity data from medicinal chemistry literature and patent sources. If available, activity data from ChEMBL were extracted at two different confidence levels, medium and high confidence, as specified in the Materials and Methods section. Fortunately, most of the 126 MTLs were detected in ChEMBL. Medium confidence data were obtained for 122 MTLs, covering a total of 979 targets, and high confidence data for 120 MTLs and 626 targets. 

**Figure 3:**
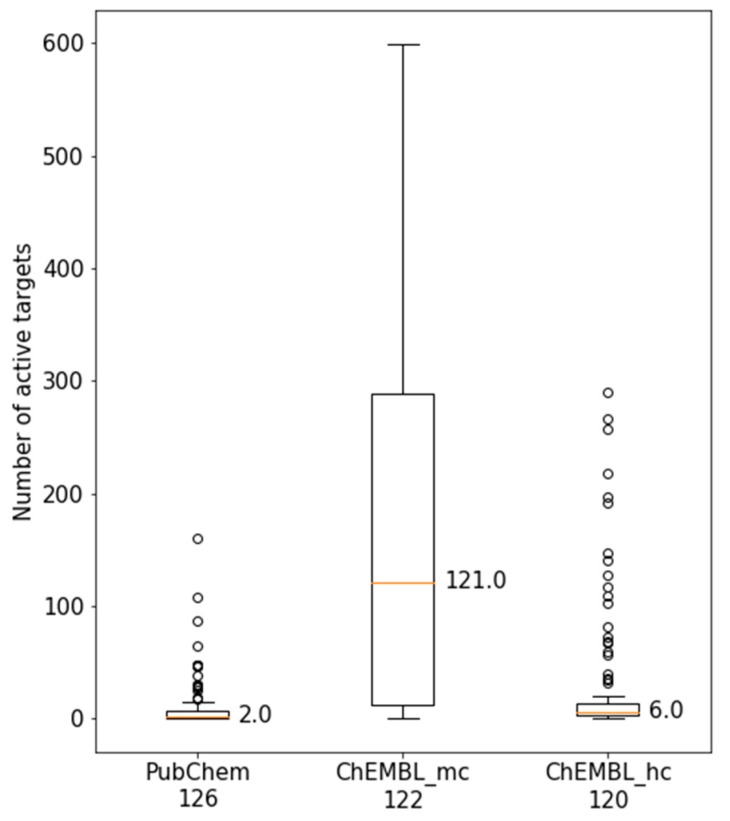
Comparison of multitarget ligand (MTL) target annotations. Boxplots report the distributions of active targets of MTLs on the basis of PubChem or ChEMBL medium confidence (mc) and high confidence (hc) activity data (see Materials and Methods for details). The number of MTLs in PubChem and ChEMBL slightly varied and is reported below each plot.

Figure 3 compares the activity of MTLs from PubChEM with ChEMBL at both data confidence levels. For high confidence activity data from ChEMBL, the distribution was also narrow, similar to the one observed for PubChem assays. However, the median value on the basis of ChEMBL data increased from 2 targets per MTL (PubChem) to 6 targets, which represented a notable increase in promiscuity. In sharp contrast to the other distributions, for ChEMBL medium confidence data, a wide distribution was observed, resulting in a large increase of the median value from 6 to 121 targets per MTL. Medium confidence data, as defined herein, require firm evidence for direct ligand-target interactions (resulting from direct binding or inhibition assays as opposed to, for example, reporter gene assays), but do not impose further constraint or confidence criteria on activity annotations. In contrast to screening data, ChEMBL data primarily originates from medicinal chemistry literature and patent sources and thus does not contain test frequency information. However, the results obtained for medium confidence activity data substantially departed from those for both PubChem assays and high confidence ChEMBL data, indicating that the majority of MTLs would be activity against more than 100 targets, which is questionable at best, if not unrealistic, as further investigated below. 

### 2.5. Confidence Analysis

**Figure 4:**
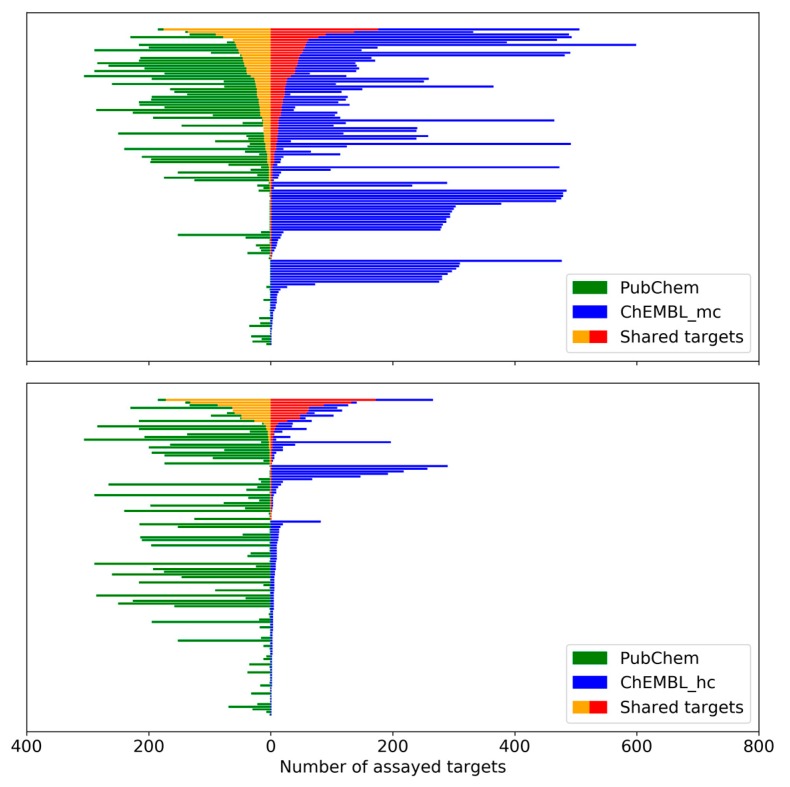
PubChem and ChEMBL targets of MTLs. For each MTL, the number of assayed PubChem targets (green) is compared to the number of reported ChEMBL targets (blue) on the basis of medium confidence (mc, top) and high confidence (hc, bottom) activity data. The number of targets shared by Pubchem and ChEMBL is highlighted (yellow and red, respectively).

For each MTL, the number of targets it was tested against in screening assays was compared to the number of targets reported in ChEMBL and the overlap between tested and targets with reported activity was determined. Figure 4 shows the results for medium and high confidence ChEMBL activity data. There was limited overlap between targets from PubChEM assays and annotated ChEMBL targets. For about half of the MTLs, no overlap was detected. However, for 89 MTLs (medium confidence data) and 46 MTLs (high confidence data) common targets were available. For a subset of these MTLs, there were large numbers of shared targets, shown in Figure 2. 

For MTLs with target overlap, it was possible to examine the consistency of screening results with target annotations from medicinal chemistry by determining the number of targets with (ChEMBL active, PubChem inactive) annotations. The results are reported in Table 1. On the basis of medium and high confidence activity data, 23 and 20 MTLs, respectively, were active in screening assays against all targets shared with ChEMBL, thus having consistent target annotations. However, on the basis of medium confidence data, 66 MTLs were inactive against 1 or more targets in screening assays they were reported to be active against in ChEMBL, including 48 MTLs with more than 2 inconsistent target annotations. For high confidence activity data, 26 of 46 MTLs had inconsistent target annotations, including 13 compounds with more than 2 inconsistencies. Hence, for MTLs with shared targets, inconsistent target annotations were frequently observed. 

**Figure 5:**
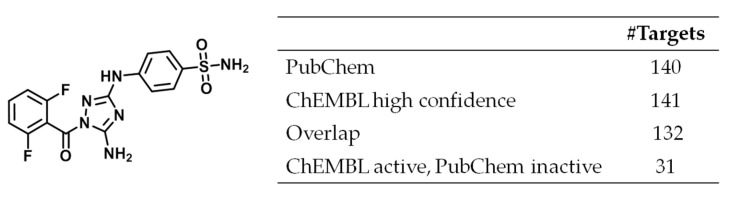
Highly promiscuous MTL. Shown is a compound (PubChem_CID 5330790, CHEMBL191003) with activity against 141 ChEMBL targets (high confidence data) that was tested against 132 of these targets in screening assays and reported to be active against 101 targets.

Only few crystallographic MTL targets were overlapping with PubChEM, i.e., structurally characteried targets were essentially not used in available screening assays. Biological screens are typically expected to be prone to false positives but MTLs were frequently inactive in screening assays for targets they reported to be active against in ChEMBL. However, apparent data inconsistency could also be resolved in a number of instances by carefully considering data sources. An example is provided in Figure 5 that shows a highly promiscuous MTL with reported activity against 141 ChEMBL targets on the basis of high confidence data. This compound was also tested against 140 PubChem targets. The overlap between PubChem and ChEMBL targets was 132 targets, which included 31 targets against which the compound was reported to be inactive in PubChem. All ChEMBL potency annotations were dissociation constant (K_d_) values and covered a range of 3.1 µM ≤ K_d_ ≤ 9.2 µM. Inconsistent target annotations exclusively resulted from a single PubChem profiling assay where compounds with a K_d_ > 3 µM were classified as inactive, hence explaining the apparent discrepancy. Although there was experimental agreement in this case for the most part, varying analysis and classification criteria led to apparent inconsistency. When activity data are explored on a large scale, e.g., in systematic compound promiscuity analysis, it is often not feasible to trace individual data sources, which likely gives rise to complications, as illustrated here. This aspect must be taken into consideration when judging promiscuity estimates. 

### 2.6. Exploring Analog Space

**Figure 6:**
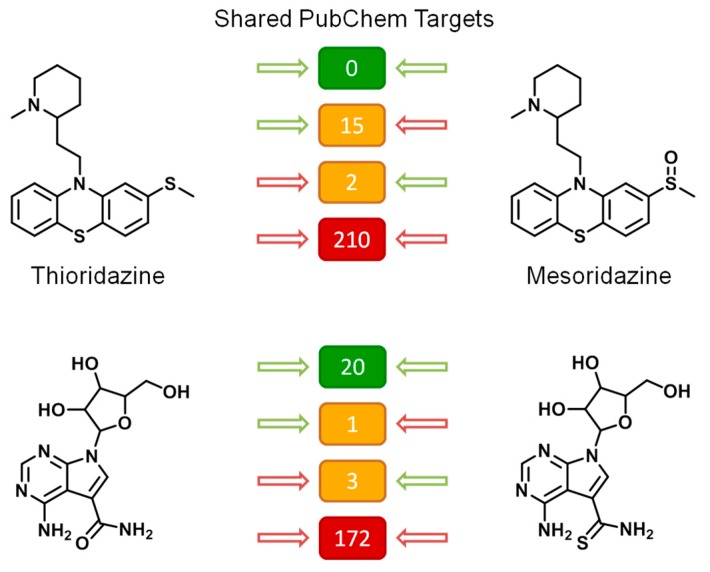
Exemplary MTL analogs. For two MTLs (left), exemplary structural analogs are shown (right). In the center, the number of shared PubChem targets is reported against which both compounds were active (green) and inactive (red) or against which only one or the other compound was active (orange, with green/red arrows).

Structural analogs of active compounds have a high probability to display similar activities. Accordingly, activity analysis can be further extended and refined by taking analogs of MTLs into consideration. Therefore, a systematic search for MTL analogs was carried out in PubChem by applying the matched molecular pair (MMP) formalism. For a subset of 46 MTLs, a total of 263 MMP relationships were detected with other PubChem compounds tested against human targets, identifying 233 structural analogs. Table 2 reports the number of targets these MTL analogs were active against. Interestingly, 111 of 233 MTL analogs were consistently inactive in all screening assays they were tested in. In addition, 72 were active against a single target, 15 against 2, and only 35 against more than 2 targets. Hence, the majority of MTL analogs were non-promiscuous on the basis of screening data, providing evidence for predominantly low promiscuity of MTL-like compounds, consistent with observations made for many MTLs, as discussed above. However, there were notable exceptions. Figure 6 below shows exemplary MTLs and analogs with different activity profiles. 

At the top in Figure 6, two drugs are shown, thioridazine, a structurally confirmed MTL used as an anti-psychotic agent, with related activity against dopamine, serotonin, and histamine receptors, and its close structural analog mesoridazine, with similar activity and therapeutic use. These compounds were tested against 227 shared targets and were inactive against 210 of them. Notably, there was no target both compounds were active against. Rather, thioridazine was active against 15 targets mesoridazine was inactive against and activity of mesoridazine was detected for two targets against which thioridazine was inactive. Hence, although there was consistent inactivity of both compounds against many targets, the activity profiles of these analogs differed significantly, with no shared activity, not even against a single target.

At the bottom in Figure 6, a guanosine-based MTL (left) is shown together with a close structural analog (right). These two compounds were tested against 196 shared targets. They were consistently inactive against 172 and active against 20 shared targets. There was 1 target against which the MTL was active but not its analog and 3 other targets against which only the analog was active. Thus, the activity profiles of these compounds over nearly 200 investigated targets were very similar, also lending credence to the screening data. Nucleoside derivatives are likely to be promiscuous, given the many functions of nucleosides in biological systems, in accord with the observations made. The comparisons in Figure 6 also show how analogs can be used to assess the consistency of activity annotations of compounds of interest.

### 2.7. Conclusions

Identifying MTLs on the basis of X-ray structures of ligand-target complexes ensured the availability of high-quality starting points for a comparative assessment of activity profiles of promiscuous chemical entities on the basis of different data sources and confidence criteria. Initially, we concentrated on the question how confirmed MTLs act across biological screens. A considerable number of 126 confirmed MTLs was detected in biological screening assays and further analysed. The promiscuity of these MTLs in screens was generally limited including large numbers of assays in which these compounds were inactive, with only few exceptions. This was an important finding because it clearly indicated ‘specificity’ of multitarget engagement and the absence of widely distributed unspecific effects. In addition, the results indicated that screens provided a meaningful source for activity data when large (statistically relevant) ensembles of target-based screening assays were considered. For activity data from the medicinal chemistry literature, test frequency information is typically unavailable. On the basis of high confidence data from ChEMBL, an increase in MTL promiscuity was observed compared to screening data; another interesting and unexpected finding. However, overall similar conclusions were drawn about MTL activity distributions for these data sources. By contrast when medium confidence activity data from ChEMBL were used an inflation of putative target annotations of MTLs was observed, suggesting that most MTLs would be active against more than 100 targets, which was unrealistic. Hence, this comparison strongly reinforced the need to base promiscuity exploration on activity data of highest possible confidence. In general, there was limited target overlap between activity data from currently available screening assay and medicinal chemistry, suggesting complementary use of these data sources for all practical purposes. A major source of data and activity profile inconsistency was that MTLs were frequently inactive in screening assays against targets for which activity was reported in the ChEMBL. In some instances, these discrepancies were found to be a consequence of applying different criteria for data curation and activity assignments. Thus, both experimental variance and applied data selection criteria are expected to contribute to inconsistencies detected herein. Finally, extending the analysis to structural analogs of MTL provided corroborating insights. The finding that close analogs of MTLs were also inactive in large numbers of assays –just as observed for MTLs– was reassuring. On the other hand, analogs displayed not only similar activity profiles but also frequent inconsistencies in target annotations compared to their MTL counterparts. Hence, the identification of structural analogs of compounds on interest provides meaningful controls for activity profile analysis. 

## 3. Materials and Methods

### 3.1. X-Ray Structures

Structures of ligand-target complexes with human proteins and single non-covalently bound compounds were extracted from the RCSB Protein Data Bank (PDB) (accessed Nov. 2019) [13]. To each qualifying PDB entry, UniProt IDs [14] and protein family annotations were added using BioServices (accessed on November 2019) [15]. 

### 3.2. Crystallographic Ligands

Information for ligands from X-ray structures was extracted from ‘macromolecular crystallographic information files’ (mmcif) available in PDBe (accessed on November 2019) [16]. Ligands denoted as ‘obsolete’ were discarded. In addition, peptides, saccharides, and polymers were omitted. Furthermore, ChEBI [17] was used to eliminate solvent and buffer molecules as well as metabolites. Moreover, ligands were required to have a molecular weight (MW) of 300 < MW < 900 Da and at least 1 numerically defined potency annotations of at least 10 µM (pK_i_, pK_d_, pIC_50_ ≥ 5) reported in ChEMBL (version 25) [9] or PDBbind (version 2018) [18]. SMILES representations [19] of ligands were standardised with the aid of the OEChem toolkit [20] and MW was calculated from SMILES using RDKit [21]. From X-ray ligands meeting these criteria, MTLs were extracted. 

### 3.3. Biological Screening Data 

From PubChem BioAssay [8] (accessed on June 2017), assays with human target annotations designated as ‘chemical screen’ with ‘no hold’ were collected. Assays with missing compound-target interactions, missing or inconsistent gene identifiers (GIs), and non-panel screens associated with multiple GIs were discarded. Furthermore, activity data imported from ChEMBL into PubChem were omitted. Each GI number was mapped to a single UniProt ID. If multiple IDs were available, preference was given to a ‘reviewed’ entry. MTLs were then mapped to accepted assays. Experimental outcomes (‘active’, ‘inactive’) were recorded as unique compound-target interactions, provided all measurements for a given pairing were consistently active or inactive. Otherwise, no interaction was recorded. 

### 3.4. Medicinal Chemistry Data 

MTLs present in PubChem were also mapped to ChEMBL. Here, two activity data confidence levels were distinguished [6]. ChEMBL medium confidence data contained human target annotations reported as direct compound-target interactions (target relationship type ‘D’) with a ChEMBL confidence score of 9. For high confidence data, additional criteria were applied. The target type was required to be a ‘single protein’ and only numerically specified measurements (‘=’) were accepted including equilibrium constant (K_i_), half maximal inhibitory concentration (IC_50_), or dissociation constant (K_d_) values with nanomolar units [6]. 

### 3.5. Analog Search 

For MTLs, a search for structural analogs was carried out in PubChem compounds assayed against human targets. Therefore, MMPs formed by an MTL and PubChem compounds were systematically identified [22]. An MMP is defined as a pair of compounds that only differ by a chemical modification at a single site [22]. For systematic generation of pairs of structural analogs, the algorithm by Hussain and Rea [22] was applied. This algorithm fragments compounds through iterative deletion of exocyclic single bonds and stores core structure (key) and substituent (value) fragments in an index table. Our in-house implementation requires keys to be at least twice the size of values and enumerates MMPs having identical keys and value fragments that are permitted to differ in size by at most eight non-hydrogen atoms. Candidate analogs with assay interference potential [23,24] or other possible chemical liabilities [25] were detected and not further considered. 

## Figures and Tables

**Table 1:** Consistency of target annotations. For MTLs, target annotations in ChEMBL and PubChem were examined for consistency. For each MTL, inconsistent annotations (ChEMBL active; PubChem inactive) on the basis of ChEMBL medium confidence (mc) and high confidence (hc) activity data were determined. MTLs with different numbers of inconsistent annotations are reported.

Number of Inactive Annotations in Overlap	Number of MTLs	
	ChEMBL_mc	ChEMBL_hc
0	23	20
1	10	8
2	8	5
>2	48	13
Number of MTLs with target overlap	89	46

**Table 2:** Target count for MTL analogs. For MTLs, a systematic search for structural analogs was carried out in PubChem. The search identified 233 MTL analogs, for which the number of targets they were active against is reported.

Number of Targets	Number of Analogs
0	111
1	72
2	15
>2	35
